# Outcomes in KEYNOTE-355 after chemotherapy discontinuation before pembrolizumab discontinuation and with immune-mediated adverse events

**DOI:** 10.1038/s41523-026-00971-8

**Published:** 2026-06-26

**Authors:** Hope S. Rugo, David W. Cescon, Peter Schmid, Zbigniew Nowecki, Seock-Ah Im, Mastura Md Yusof, Carlos Gallardo, Hiroji Iwata, Oleg Lipatov, Felipe Cruz, Carlos Henrique Barrios, Roberto Hegg, Sherene Loi, Maria Gion, Marco Torregroza Otero, Péter Árkosy, Rafal Tarnawski, Lili Yao, Jaime Mejia, Wilbur Pan, Javier Cortés

**Affiliations:** 1 City of Hope Comprehensive Cancer Center, Duarte, CA USA; 2 Princess Margaret Cancer Centre, University Health Network, University of Toronto, Toronto, ON Canada; 3 Barts Cancer Institute, Centre for Experimental Cancer Medicine, Queen Mary University of London, London, UK; 4 Breast Cancer and Reconstructive Surgery Department, Maria Sklodowska-Curie National Research Institute of Oncology, Warsaw, Poland; 5 Seoul National University Hospital, Seoul National University College of Medicine, Cancer Research Institute, Seoul National University, Seoul, Republic of Korea; 6 Hospital PICASO, Petaling Jaya, Malaysia; 7 Bradford Hill Research Center, Santiago, Chile; 8 Nagoya City University, Nagoya, Aichi Japan; 9 Republican Clinical Oncology Dispensary of the Ministry of Public Health of Bashkortostan Republic, Ufa, Russia; 10 Instituto Brasileiro de Controle do Câncer, São Paulo, Brazil; 11 Latin American Cooperative Oncology Group (LACOG), Porto Alegre, Brazil; 12 Gynecological and Breast Oncology, Clínica de Pesquisas e Centro de Estudos em Oncologia Ginecológica e Mamária Ltda., Avenida Brigadeiro Luís Antônio, São Paulo, Brazil; 13 Division of Cancer Research, Peter MacCallum Cancer Centre, Melbourne, and The Sir Peter MacCallum Department of Medical Oncology, University of Melbourne, Parkville, VIC Australia; 14 Hospital Universitario Ramón y Cajal, Madrid, Spain; 15 Oncomedica, Montería, Colombia; 16 Department of Oncology, University of Debrecen, Debrecen, Hungary; 17 Maria Sklodowska-Curie National Research Institute of Oncology, Gliwice, Poland; 18 Merck & Co., Inc., Rahway, NJ USA; 19 International Breast Cancer Center, Pangaea Oncology, Quiron Group, Barcelona, Spain; IOB Madrid, Hospital Beata Maria Ana, Madrid, Spain; Universidad Europea de Madrid, Faculty of Biomedical and Health Sciences, Department of Medicine, Madrid, Spain

**Keywords:** Cancer, Immunology, Oncology

## Abstract

Pembrolizumab plus chemotherapy significantly improved progression-free survival (PFS) and overall survival (OS) versus chemotherapy in the phase 3 randomized KEYNOTE-355 study in participants with previously untreated, advanced triple-negative breast cancer (TNBC) with PD-L1 combined positive score (CPS) ≥ 10. Among participants with clinical benefit from pembrolizumab plus chemotherapy, discontinuation of chemotherapy before pembrolizumab for any reason was associated with similar median PFS and OS to that in the overall population. Additionally, we further characterized immune-mediated adverse events (AEs) in KEYNOTE-355. Most immune-mediated AEs were manageable with treatment interruption and supportive care. In participants treated with pembrolizumab plus chemotherapy with immune-mediated AEs, there appeared to be numerical improvement in efficacy. This analysis provides additional information on the use of pembrolizumab plus chemotherapy as standard-of-care therapy in patients with previously untreated, advanced TNBC with PD-L1 CPS ≥ 10. This trial is registered at ClinicalTrials.gov (NCT02819518; registration date, June 28, 2016).

## Introduction

In the phase 3 KEYNOTE-355 study (ClinicalTrials.gov, NCT02819518), pembrolizumab plus chemotherapy (investigator’s choice of nanoparticle albumin-bound paclitaxel, paclitaxel, or gemcitabine plus carboplatin) led to statistically significant and clinically meaningful improvements in the primary endpoints of progression-free survival (PFS) and overall survival (OS) when compared with placebo plus chemotherapy in participants with previously untreated, locally recurrent inoperable or metastatic triple-negative breast cancer (TNBC) with tumors expressing programmed cell death ligand 1 (PD-L1) with a combined positive score (CPS) ≥ 10^1,2^. Among these participants, the median PFS at the second interim analysis (IA2; data cutoff: December 11, 2019) was 9.7 months with pembrolizumab plus chemotherapy versus 5.6 months with placebo plus chemotherapy (hazard ratio [HR], 0.65 [95% CI, 0.49‒0.86; 1-sided *P* = 0.0012])^1^. OS at the final analysis (data cutoff: June 15, 2021) was 23.0 months and 16.1 months in the respective groups (HR, 0.73 [95% CI, 0.55‒0.95; 2-sided *P* = 0.0185])^2^.

The safety profile observed at the final analysis of KEYNOTE-355 was generally consistent with the known safety profiles of the individual agents^2^. Treatment-related adverse events (AEs), as reported by the investigators, occurred in 96.3% of participants treated with pembrolizumab plus chemotherapy and 95.0% of those treated with placebo plus chemotherapy. Immune-mediated AEs of any grade were reported in 26.5% of participants treated with pembrolizumab plus chemotherapy versus 6.4% of participants treated with placebo plus chemotherapy^2^.

Based on results from KEYNOTE-355, pembrolizumab in combination with chemotherapy has been approved in several countries^3,4^ for the treatment of patients with locally recurrent unresectable or metastatic TNBC whose tumors express PD-L1 with a CPS of ≥10. Herein, we report post hoc exploratory analyses of early chemotherapy discontinuation prior to pembrolizumab and its effects on efficacy outcomes in participants with clinical benefit. We also further characterize immune-mediated AEs and their management from the KEYNOTE-355 final analysis and present post hoc exploratory assessments of associations between immune-mediated AEs and efficacy outcomes.

## Results

### Participants

At the time of data cutoff for the current analysis (June 15, 2021; ie, the final analysis), 847 participants were randomized, with 566 assigned to pembrolizumab plus chemotherapy and 281 assigned to placebo plus chemotherapy. The safety population included 843 participants, with 562 in the pembrolizumab plus chemotherapy group and 281 in the placebo plus chemotherapy group. The pembrolizumab plus chemotherapy group included 141 participants with PD-L1 CPS < 1, 421 participants with PD-L1 CPS ≥ 1 (including CPS ≥ 10), and 219 participants with PD-L1 CPS ≥ 10. As previously reported^1^, baseline demographics and disease characteristics were generally well balanced between the treatment groups. At the final analysis, the median duration of any study treatment was 6.1 (range, 0.0‒48.8) months in the pembrolizumab plus chemotherapy group and 5.3 (range, 0.0‒51.5) months in the placebo plus chemotherapy group.

In the safety population, 84 participants (14.9%) in the pembrolizumab plus chemotherapy group had AEs leading to chemotherapy discontinuation, most commonly neutropenia (1.8%), neuropathy peripheral (1.2%), neutrophil count decreased (1.1%), and peripheral sensory neuropathy (1.1%). Chemotherapy agents discontinued due to AEs were nab-paclitaxel (3.2%), paclitaxel (2.8%), gemcitabine (5.9%), and carboplatin (6.6%).

### Outcomes among participants with clinical benefit who discontinued chemotherapy before pembrolizumab

**Fig. 1:**
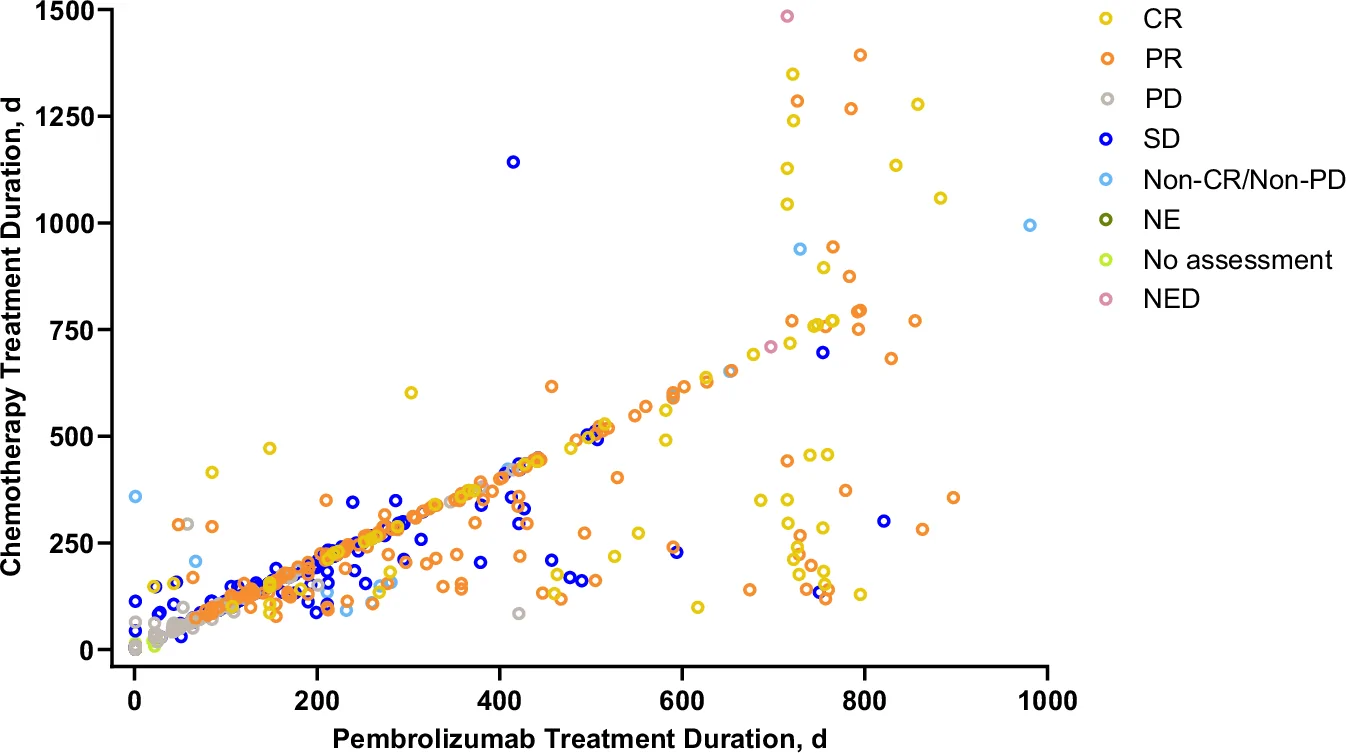
Best overall response by total pembrolizumab and chemotherapy treatment duration. CR, complete response; NE, not evaluable; NED, no evidence of disease; PD, progressive disease; PR, partial response; SD, stable disease. NED includes participants without evidence of disease at baseline and ≥1 post-baseline time point.

At data cutoff, 317 participants in the pembrolizumab plus chemotherapy group had a complete response (CR), partial response (PR), or stable disease (SD) lasting ≥24 weeks, of whom 92 (29.0%) had discontinued chemotherapy before pembrolizumab (ie, receipt of the last dose of chemotherapy >21 days before the last dose of pembrolizumab). Among these participants, the median (range) durations of pembrolizumab and chemotherapy treatment, respectively, were 14.1 (4.2‒29.5) and 6.0 (2.6‒25.3) months (Table 1). Discontinuation of chemotherapy before pembrolizumab was associated with similar median PFS and median OS to those in the overall population, irrespective of tumor PD-L1 expression level (Table 1). In the all-participants-as-treated population, participants who discontinued chemotherapy before pembrolizumab had a median PFS and OS of 14.5 (95% CI, 11.9‒20.2) months and 32.9 (95% CI, 27.4‒38.3) months, respectively, compared with 11.6 (95% CI, 9.9‒12.3) months and 26.4 (95% CI, 23.5‒29.7) months in the overall population (Table 1). Furthermore, there were participants who discontinued chemotherapy early but continued to have clinical benefit with continued pembrolizumab treatment (Fig. 1). Clinical responses by treatment duration in participants with PD-L1 CPS ≥ 1 and CPS ≥ 10 are shown in Supplementary Fig. S1.

**Table 1:** Kaplan–Meier-estimated PFS and OS in participants with clinical benefit^a^ from pembrolizumab plus chemotherapy

Analysis population	*N*	Median pembrolizumab treatment duration (range), mo	Median chemotherapy treatment duration (range), mo	Median (95% CI) PFS, mo	Median (95% CI) OS, mo
All participants as treated					
Participants who discontinued chemotherapy before pembrolizumab^b^	92	14.1 (4.2‒29.5)	6.0 (2.6‒25.3)	14.5 (11.9‒20.2)	32.9 (27.4‒38.3)
Overall	317	9.4 (0.0‒32.2)	7.9 (0.3‒45.8)	11.6 (9.9‒12.3)	26.4 (23.5‒29.7)
PD-L1 CPS ≥ 1					
Participants who discontinued chemotherapy before pembrolizumab^b^	70	15.3 (4.2‒29.5)	5.9 (2.6‒24.7)	18.7 (14.4‒NR)	34.5 (27.9‒NR)
Overall	249	9.4 (0.0‒32.2)	8.2 (1.4‒45.8)	11.7 (9.9‒13.9)	26.6 (23.5‒30.5)
PD-L1 CPS ≥ 10					
Participants who discontinued chemotherapy before pembrolizumab^b^	46	20.8 (4.9‒29.5)	6.8 (3.5‒24.7)	36.7 (17.3‒NR)	NR (34.3‒NR)
Overall	143	11.1 (0.0‒32.2)	8.5 (3.0‒45.8)	14.4 (11.3‒17.7)	34.4 (26.7‒42.5)

*CPS* combined positive score, *CR* complete response, *NR* not reached, *OS* overall survival, *PD-L1* programmed cell death ligand 1, *PFS* progression-free survival, *PR* partial response, *SD* stable disease.

^a^CR, PR, or SD lasting ≥24 weeks.

^b^Last dose of chemotherapy received >21 days before the last dose of pembrolizumab.

### Immune-mediated adverse events

**Fig. 2:**
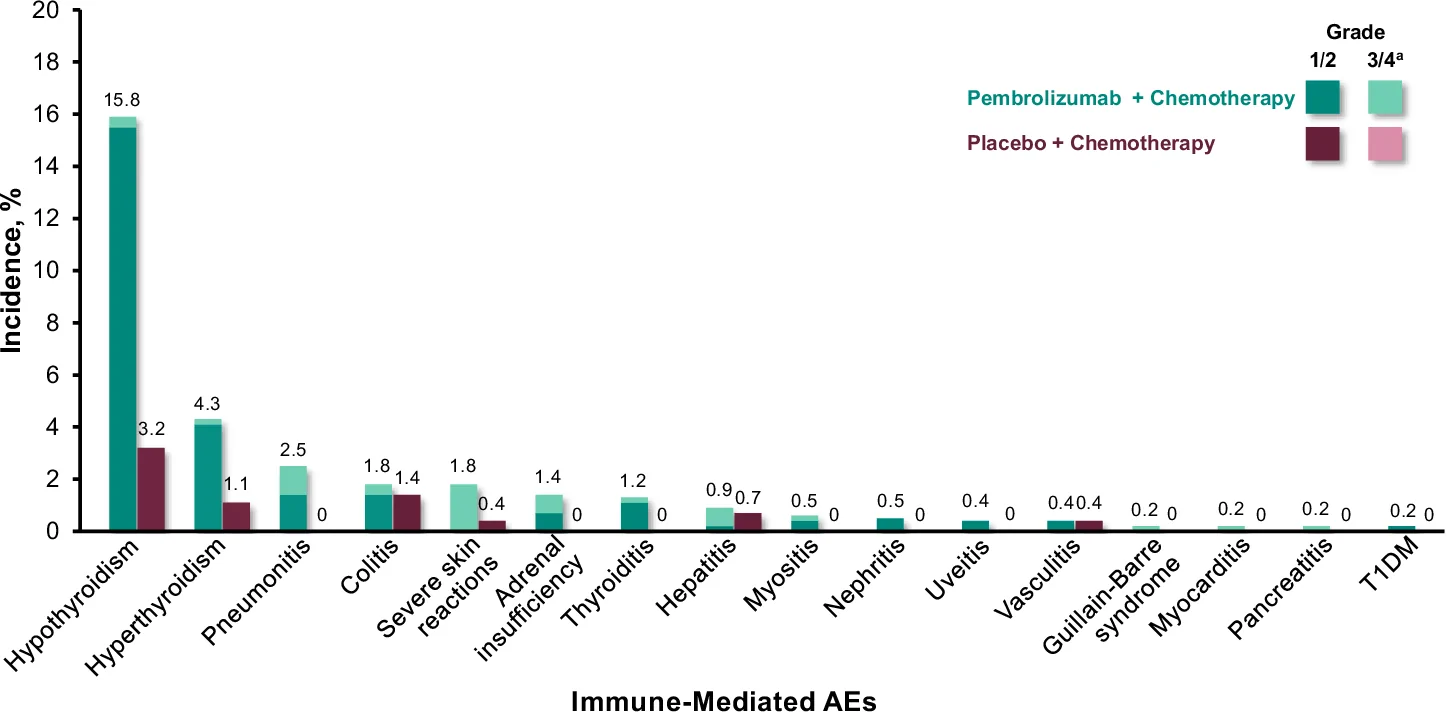
Immune-mediated AEs by grade. ^a^No grade 5 events were reported. AE, adverse event; T1DM, type 1 diabetes mellitus.

In general, pembrolizumab plus chemotherapy had a manageable immune safety profile in participants with previously untreated, advanced TNBC. Immune-mediated AEs occurred more frequently in the pembrolizumab plus chemotherapy group. As reported in the KEYNOTE-355 final analysis^2^, the overall incidence of immune-mediated AEs was higher with pembrolizumab plus chemotherapy (26.5%) than with placebo plus chemotherapy (6.4%; Table 2). Most immune-mediated AEs were grade 1 or 2 in severity (Table 2 and Fig. 2). Of 149 participants (26.5%) who experienced immune-mediated AEs in the pembrolizumab plus chemotherapy group, 119 (21.2%) had grade 1 or 2 events and 30 (5.3%) had grade 3 or 4 events; no participant had a grade 5 event. Most immune-mediated AEs were manageable with treatment interruption, corticosteroids, and/or hormone replacement and did not result in discontinuation of study treatment (Table 3 and Table 4). In the pembrolizumab plus chemotherapy group, 125 participants (22.2%) had immune-mediated AEs requiring treatment with corticosteroids. Immune-mediated AEs led to interruption of pembrolizumab in 35 participants. (2.5%; Table 3). Most immune-mediated AEs leading to pembrolizumab interruption or discontinuation were grade 2 or 3. Six participants (1.1%) discontinued chemotherapy due to immune-mediated AEs (pneumonitis, *n* = 3; Guillain-Barre syndrome, hepatitis, and hypothyroidism, *n* = 1 each). Chemotherapy agents discontinued owing to these events were nab-paclitaxel (*n* = 3), paclitaxel (*n* = 1), gemcitabine (*n* = 2), and carboplatin (*n* = 1).

**Table 2:** Summary of immune-mediated adverse events

	Pembrolizumab plus chemotherapy *n* = 562	Placebo plus chemotherapy *n* = 281
Any	149 (26.5)	18 (6.4)
Grade ½	119 (21.2)	18 (6.4)
Grade 3/4	30 (5.3)	0
Grade 5	0	0
Led to dose reduction^a^		
Chemotherapy	1 (0.2)^b^	0
Led to treatment interruption		
Pembrolizumab/placebo	35 (6.2)	2 (0.7)
Chemotherapy	34 (6.0)	4 (1.4)
Led to discontinuation of any drug		
Pembrolizumab/placebo	14 (2.5)	0
Chemotherapy	6 (1.1)	0

Data presented are *n* (%) of participants; participants may have experienced ≥1 immune-mediated adverse event.

^a^Dose reduction was not allowed for pembrolizumab or placebo.

^b^Due to hepatitis.

**Table 3:** Immune-mediated adverse events resulting in pembrolizumab or placebo discontinuation or interruption

	Discontinuation		Interruption	
	Pembrolizumab plus chemotherapy *n* = 562	Placebo plus chemotherapy *n* = 281	Pembrolizumab plus chemotherapy *n* = 562	Placebo plus chemotherapy *n* = 281
Any	14 (2.5)	0	35 (6.2)	2 (0.7)
Pneumonitis	7 (1.2)	0	9 (1.6)	0
Hypothyroidism	0	0	7 (1.2)	1 (0.4)
Severe skin reactions	0	0	7 (1.2)	1 (0.4)
Colitis	1 (0.2)	0	4 (0.7)	0
Adrenal insufficiency	1 (0.2)	0	4 (0.7)	0
Nephritis	0	0	2 (0.4)	0
Hepatitis	2 (0.4)	0	1 (0.2)	0
Hyperthyroidism	0	0	1 (0.2)	0
Thyroiditis	0	0	1 (0.2)	0
Type 1 diabetes mellitus	0	0	1 (0.2)	0
Pancreatitis	1 (0.2)	0	0	0
Myocarditis	1 (0.2)	0	0	0
Guillain-Barre syndrome	1 (0.2)	0	0	0

Data presented are *n* (%) of participants.

**Table 4:** Time to onset and management of the most common^a^ immune-mediated adverse events

	Pembrolizumab plus chemotherapy *n* = 562	Placebo plus chemotherapy *n* = 281
Hypothyroidism		
Participants with event, *n* (%)	89 (15.8)	9 (3.2)
Median time to onset (range), d	105 (19–707)	80 (20–531)
Treated with thyroid replacement, *n*	70	6
Hyperthyroidism		
Participants with event, *n* (%)	24 (4.3)	3 (1.1)
Median time to onset (range), d	53.5 (20–209)	85 (78–317)
Pneumonitis^b^		
Participants with event, *n* (%)	14 (2.5)	0
Median time to onset (range), d	99.5 (29–288)	–
Treated with corticosteroids, *n*	11	–
Colitis		
Participants with event, *n* (%)	10 (1.8)	4 (1.4)
Median time to onset (range), d	232.5 (23−469)	94.5 (24−260)
Treated with corticosteroids, *n*	7	1
Severe skin reactions		
Participants with event, *n* (%)	10 (1.8)	1 (0.4)
Median time to onset (range), d	91 (4−283)	125 (125−125)
Treated with corticosteroids, *n*	10	0
Adrenal insufficiency		
Participants with event, *n* (%)	8 (1.4)	0
Median time to onset (range), d	242 (75–828)	–
Treated with hormone replacement, *n*	8	–
Thyroiditis		
Participants with event, *n* (%)	7 (1.2)	0
Median time to onset (range), d	44 (22−270)	–
Treated with thyroid replacement, *n*	3	–
Hepatitis		
Participants with event, *n* (%)	5 (0.9)	2 (0.7)
Median time to onset (range), d	85 (6−339)	28 (7−49)
Treated with corticosteroids, *n*	5	0

“+” indicates the episode was not recovered by the time of the data cutoff or date of death.

^a^Occurring in ≥5 participants in the pembrolizumab plus chemotherapy group.

^b^Median episode duration based on Kaplan–Meier method for censored data: pembrolizumab plus chemotherapy, 56 (range, 10–1267 +) days.

The most frequently reported immune-mediated AEs in the pembrolizumab plus chemotherapy group were hypothyroidism (15.8%), hyperthyroidism (4.3%), and pneumonitis (2.5%; Table 4 and Fig. 2); 12 participants (2.1%) had both hypothyroidism and hyperthyroidism. The median time to onset varied for each immune-mediated AE, with most of those in the pembrolizumab plus chemotherapy group occurring after the first 3 months of treatment and within 1 year (Table 4). The median time to onset of the first immune-mediated AE was 85 (range, 4‒828) days in the pembrolizumab plus chemotherapy group.

Infusion reactions occurred in 21 participants (3.7%; grade 3 or 4 in 3 participants [0.5%]) in the pembrolizumab plus chemotherapy group and 14 participants (5.0%; no grade ≥3 events) in the placebo plus chemotherapy group. Ten participants in the pembrolizumab plus chemotherapy group and 8 participants in the placebo plus chemotherapy group were treated with corticosteroids for infusion reactions. The median (range) time to onset of an infusion reaction was 147 (1‒729) and 110 (4‒467) days, respectively.

### Outcomes among participants who experienced immune-mediated adverse events

A total of 149 participants receiving pembrolizumab plus chemotherapy experienced an immune-mediated AE. The median duration of treatment with pembrolizumab was numerically longer among participants who experienced immune-mediated AEs compared with the overall population (8.8 vs 5.6 months; Table 5). The median duration of treatment with chemotherapy was also numerically longer among participants who experienced immune-mediated AEs than in the overall population (7.2 vs 5.1 months; Table 5). In participants with immune-mediated AEs, efficacy in the pembrolizumab plus chemotherapy arm appeared to be numerically better, with a median PFS and OS of 9.7 (95% CI, 8.0‒11.6) months and 23.9 (95% CI, 20.6‒28.6) months, respectively, compared with 7.5 (95% CI, 6.3‒7.7) months and 17.2 (95% CI, 15.3‒19.0) months in the overall population (Table 5). This pattern was also observed in the PD-L1 CPS ≥ 1 and CPS ≥ 10 subgroups (Table 5).

**Table 5:** Kaplan–Meier-estimated PFS and OS in participants treated with pembrolizumab plus chemotherapy who had immune-mediated AEs

Analysis population	*N*	Median pembrolizumab treatment duration (range), mo	Median chemotherapy treatment duration (range), mo	Median (95% CI) PFS, mo	Median (95% CI) OS, mo
All participants as treated					
Participants who had immune-mediated AEs	149	8.8 (0.0–29.0)	7.2 (0.3–41.7)	9.7 (8.0–11.6)	23.9 (20.6–28.6)
Overall	562	5.6 (0.0–32.2)	5.1 (0.0–48.8)	7.5 (6.3-7.7)	17.2 (15.3–19.0)
PD-L1 CPS ≥ 1					
Participants who had immune-mediated AEs	109	8.8 (0.7–29.0)	7.3 (1.0–34.8)	9.8 (8.0–16.5)	26.3 (21.2–32.7)
Overall	421	5.9 (0.0-32.2)	5.1 (0.0-48.8)	7.6 (6.6-8.0)	17.6 (15.5–19.5)
PD-L1 CPS ≥ 10					
Participants who had immune-mediated AEs	64	10.4 (0.8–29.0)	8.4 (2.3–34.8)	11.8 (9.5–NR)	35.6 (26.3–NR)
Overall	219	7.6 (0.0-32.2)	5.8 (0.0-45.8)	9.7 (7.6-11.3)	22.8 (18.8–26.2)

*AE* adverse event, *CPS* combined positive score, *NR* not reached, *OS* overall survival, *PD-L1* programmed cell death ligand 1, *PFS* progression-free survival.

## Discussion

Our results suggest that patients with locally recurrent inoperable or metastatic TNBC who have clinical benefit with first-line treatment with pembrolizumab plus chemotherapy do not have adverse outcomes if chemotherapy is discontinued early. There was no evidence of an adverse effect on PFS or OS among participants who had CR, PR, or SD lasting ≥24 weeks and discontinued chemotherapy before discontinuing pembrolizumab, independent of PD-L1 expression level (Table 1). Of note, median PFS and OS were numerically longer in participants who had tumors with PD-L1 CPS ≥ 10 than in all participants as treated and those with tumors with PD-L1 CPS ≥ 1 (Table 1). This finding may be clinically relevant because pembrolizumab plus chemotherapy is approved in the United States and European Union for the treatment of patients with locally recurrent unresectable or metastatic TNBC with PD-L1 CPS ≥ 10^3,4^.

As previously reported, the immune safety profile of pembrolizumab plus chemotherapy in participants with previously untreated, locally advanced inoperable or metastatic TNBC was generally consistent with the known safety profiles of pembrolizumab and the individual chemotherapy regimens^5–7^. Most immune-mediated AEs were manageable using treatment interruption and supportive care (ie, corticosteroids, hormone replacement). The most frequently reported immune-mediated AEs associated with pembrolizumab plus chemotherapy (Table 4) were consistent with those most commonly reported for pembrolizumab monotherapy in patients with metastatic TNBC^6^ and other advanced cancers^7^. Infusion reactions were uncommon and occurred more frequently in the placebo plus chemotherapy group than in the pembrolizumab plus chemotherapy group; most were low grade and manageable.

The occurrence of immune-mediated AEs did not adversely affect efficacy outcomes in participants who received pembrolizumab plus chemotherapy, irrespective of PD-L1 CPS status (Table 5). Indeed, estimates of median OS and PFS were consistently numerically longer among participants receiving pembrolizumab plus chemotherapy who experienced immune-mediated AEs compared with those in the overall study population. However, it is important to note that treatment duration was also longer among participants who experienced immune-mediated AEs and that this may be related to both the likelihood of experiencing an immune-mediated AE and the longer OS and PFS in that population. Improved efficacy outcomes with immune checkpoint inhibitors have been observed in patients who experienced immune-related AEs compared with those who did not across various cancers^8–11^; however, these findings require prospective confirmation.

These analyses have limitations. In particular, the evaluation of outcomes in participants who discontinued chemotherapy before pembrolizumab and the evaluation of associations between immune-mediated AEs and outcomes were retrospective exploratory analyses and the results should be interpreted cautiously. Moreover, the follow-up period for AEs in this study was 30 days (90 days for serious AEs) and any immune-mediated AEs that occurred after this period were not collected as part of the study. It is important to note that these analyses were descriptive (ie, no alpha was assigned) and they were not controlled for multiplicity and, therefore, further investigation is needed to fully understand this relationship.

In conclusion, these results, together with the clinical benefits previously reported^1,2^, provide additional information to support the use of pembrolizumab plus chemotherapy as a standard of care regimen in patients with previously untreated, locally recurrent inoperable or metastatic TNBC with PD-L1 expression at the cutoff of CPS ≥ 10. In patients with locally recurrent inoperable or metastatic TNBC receiving first-line treatment with pembrolizumab plus chemotherapy, the continuation of pembrolizumab treatment should be considered for those who discontinue chemotherapy for reasons other than disease progression and should be discussed with the patient. The data presented here are based on retrospective analyses in a subset of the population enrolled in KEYNOTE-355 and need further confirmation in prospective trials. The immune safety profile of pembrolizumab plus chemotherapy in participants with TNBC was generally consistent with its known safety profile in other indications. Most immune-mediated AEs were manageable, and their occurrence did not appear to adversely affect the efficacy of pembrolizumab plus chemotherapy. The potential association of immune-mediated AEs with efficacy in patients receiving pembrolizumab is unclear in the absence of adequately powered prospective studies designed to evaluate this association.

## Methods

### Study design and participants

The study design and methods of KEYNOTE-355 have been described previously^1,2^; key methods are outlined briefly here. KEYNOTE-355 (ClinicalTrials.gov, NCT02819518; date of registration, June 28, 2016) was a phase 3, randomized, double-blind, placebo-controlled study that enrolled participants in 29 countries. Eligible participants were aged ≥18 years and had previously untreated, centrally confirmed, locally recurrent inoperable or metastatic TNBC, measurable disease according to Response Evaluation Criteria in Advanced Solid Tumors (RECIST) version 1.1, an Eastern Cooperative Oncology Group performance status of 0 or 1, and adequate organ function. Participants were eligible if they had de novo metastatic TNBC or ≥6 months had elapsed between completion of curative-intent treatment for stage I‒III breast cancer and first documented disease recurrence. TNBC and PD-L1 status were centrally determined from newly obtained or archival tumor samples from a locally recurrent inoperable or metastatic site; participants were eligible to enter the trial irrespective of PD-L1 status. The study was conducted in accordance with principles of Good Clinical Practice and was approved by the appropriate institutional review boards and regulatory agencies. All participants provided written informed consent.

Participants were randomized 2:1 to receive either intravenous pembrolizumab 200 mg every 3 weeks plus chemotherapy or placebo plus chemotherapy. Chemotherapy was the investigator’s choice of 1 of the following: nab-paclitaxel 100 mg/m^2^ on days 1, 8, and 15 of every 28-day cycle; paclitaxel 90 mg/m^2^ on days 1, 8, and 15 of every 28-day cycle; or gemcitabine 1000 mg/m^2^ plus carboplatin (area under the concentration-time curve 2) on days 1 and 8 of every 21-day cycle. Treatment was continued for up to 35 cycles of pembrolizumab or placebo (chemotherapy was continued at the investigator’s discretion) or until disease progression, unacceptable toxic effects, withdrawal of consent, or physician decision. The sponsor, investigators, other study site staff, and participants were blinded to treatment assignment and tumor PD-L1 status.

### Endpoints

The dual primary endpoints of the original study were PFS per RECIST version 1.1 by blinded independent central review (BICR) and OS in participants whose tumors expressed PD-L1 at the CPS cutoff of ≥10 or ≥1 and in the intention-to-treat (ITT) population. Secondary efficacy endpoints included objective response rate, duration of response, and disease control rate. Safety was a secondary endpoint and was assessed among all participants who had received ≥1 dose of pembrolizumab, placebo, or chemotherapy.

### Assessments

Imaging for the assessment of clinical response was performed at baseline and every 8 weeks until week 24, then every 9 weeks during the first year and every 12 weeks thereafter. Tumor response was assessed per RECIST version 1.1 by BICR. Baseline PD-L1 expression, centrally assessed using PD-L1 IHC 22C3 pharmDx (Agilent Technologies, Carpinteria, CA, USA), was characterized according to CPS, which was defined as the number of PD-L1‒positive cells (tumor cells, lymphocytes, and macrophages) divided by the total number of tumor cells multiplied by 100.

Adverse events were monitored throughout the study and for 30 days (90 days for serious AEs) after discontinuation of study treatment. The severity of AEs was graded according to National Cancer Institute Common Terminology Criteria for Adverse Events version 4.0. Immune-mediated AEs were determined on the basis of a prespecified list of Medical Dictionary for Regulatory Activities terms intended to capture the known risks of pembrolizumab. These were considered irrespective of whether they were deemed to be attributable to study treatment by the investigator. The time to AE onset and treatment of AEs was evaluated for the most common immune-mediated AEs and infusion reactions.

### Analysis and statistical considerations

The current analyses evaluated PFS and OS in participants who had clinical benefit and discontinued chemotherapy before pembrolizumab, examined immune-mediated AEs and their management from the final analysis, and evaluated PFS and OS in participants who experienced previous immune-mediated AEs.

Using the Kaplan–Meier method, BICR-assessed PFS (per RECIST version 1.1) and OS were assessed in participants in the pembrolizumab plus chemotherapy treatment group who had achieved a best overall response of CR, PR, or SD lasting ≥24 weeks and received their last dose of chemotherapy >21 days before their last dose of pembrolizumab and in participants who had experienced ≥1 immune-mediated AE. HRs with 95% CIs were calculated by a Cox proportional hazards model with the Efron method of tie handling.

The incidences of AEs, immune-mediated AEs, and infusion reactions were compared between the pembrolizumab plus chemotherapy and placebo plus chemotherapy treatment groups using descriptive analyses only. Safety was evaluated in the all-participants-as-treated population (ie, all randomized participants who received ≥1 dose of study treatment, analyzed according to treatment received).

## Supplementary information

Supplementary information

Supplementary information

## Data Availability

Merck Sharp & Dohme LLC, a subsidiary of Merck & Co., Inc., Rahway, NJ, USA (MSD) is committed to providing qualified scientific researchers access to anonymized data and clinical study reports from the company’s clinical trials for the purpose of conducting legitimate scientific research. MSD is also obligated to protect the rights and privacy of trial participants and, as such, has a procedure in place for evaluating and fulfilling requests for sharing company clinical trial data with qualified external scientific researchers. The MSD data sharing website (available at: https://externaldatasharing-msd.com/) outlines the process and requirements for submitting a data request. Applications will be promptly assessed for completeness and policy compliance. Feasible requests will be reviewed by a committee of MSD subject matter experts to assess the scientific validity of the request and the qualifications of the requestors. In line with data privacy legislation, submitters of approved requests must enter into a standard data-sharing agreement with MSD before data access is granted. Data will be made available for request after product approval in the US and EU or after product development is discontinued. There are circumstances that may prevent MSD from sharing requested data, including country- or region-specific regulations. If the request is declined, it will be communicated to the investigator. Access to genetic or exploratory biomarker data requires a detailed, hypothesis-driven statistical analysis plan that is collaboratively developed by the requestor and MSD subject matter experts; after approval of the statistical analysis plan and execution of a data-sharing agreement, MSD will either perform the proposed analyses and share the results with the requestor or will construct biomarker covariates and add them to a file with clinical data that is uploaded to an analysis portal so that the requestor can perform the proposed analyses.
