# Supplementary material for: Outcomes in KEYNOTE-355 after chemotherapy discontinuation before pembrolizumab discontinuation and with immune-mediated adverse events
Source: NPJ Breast Cancer. 2026 Jun 26;12:137. doi: 10.1038/s41523-026-00971-8 (PMC13677730; doi:10.1038/s41523-026-00971-8)
Supplement: Supplementary file 1 [file 41523_2026_971_MOESM1_ESM.docx]

# SUPPLEMENTARY INFORMATION

Supplementary Figure S1. Best overall response by total pembrolizumab and chemotherapy treatment duration in participants with (A) tumor PD-L1 CPS ≥1 and (B) tumor PD-L1 CPS ≥10. CPS, combined positive score; CR, complete response; NE, not evaluable; NED, no evidence of disease; PD, progressive disease; PD-L1, programmed cell death ligand 1; PR, partial response; SD, stable disease. NED includes participants without evidence of disease at baseline and ≥1 post-baseline time point.

**A**

**B**
